# Emerging Mechanisms and Therapeutic Strategies in Dilated Cardiomyopathy

**DOI:** 10.3390/biomedicines14030523

**Published:** 2026-02-26

**Authors:** Linlin Wang, Chen Chen, Dao Wen Wang

**Affiliations:** 1 Division of Cardiology, Departments of Internal Medicine and Genetic Diagnosis Center, Tongji Hospital, Tongji Medical College, Huazhong University of Science and Technology, Wuhan 430030, China; wanglinlin202410@163.com (L.W.); chenchen@tjh.tjmu.edu.cn (C.C.); 2 Hubei Key Laboratory of Genetics and Molecular Mechanisms of Cardiological Disorders, Huazhong University of Science and Technology, Wuhan 430030, China; 3 Genetic Diagnosis Center, Tongji Hospital, Tongji Medical College, Huazhong University of Science and Technology, Wuhan 430030, China; 4 Sino-German Laboratory of CardioPulmonary Science, Tongji Medical College, Huazhong University of Science and Technology, Wuhan 430030, China

**Keywords:** dilated cardiomyopathy, second hits, clonal hematopoiesis, targeting therapies

## Abstract

Dilated cardiomyopathy (DCM) is a leading cause of heart failure and heart transplantation and is characterized by marked clinical and etiological heterogeneity. Recent studies have expanded the understanding of DCM from a predominantly monogenic disorder to a multifactorial disease shaped by genetic susceptibility and acquired or environmental “second hits”. Beyond rare pathogenic variants, emerging evidence highlights the contribution of clonal hematopoiesis of indeterminate potential to inflammation-driven adverse cardiac remodeling and disease progression. These secondary modifiers interact with pre-existing genetic backgrounds to amplify shared downstream pathways. In parallel, advances in mechanism-informed therapies are increasingly translating these insights into clinical practice. Beyond guideline-directed medical and device therapy, emerging approaches targeting specific molecular pathways, including sarcomeric modulators, inflammatory signaling, and gene- or cell-based interventions, illustrate a shift toward more personalized and stage-specific management of DCM and heart failure. This review aims to provide an updated overview of recent advances in the molecular mechanisms and diagnosis underlying DCM and discuss their implications for current and emerging therapeutic strategies.

## 1. Introduction

Dilated cardiomyopathy (DCM, OMIM #115200) is a heterogeneous myocardial disorder characterized by dilation of the left or both ventricles accompanied by systolic dysfunction, in the absence of identifiable causes such as coronary artery disease, hypertension, valvular heart disease, or congenital heart disease [1]. Clinical presentation is highly variable, ranging from asymptomatic stages to heart failure or sudden cardiac death (SCD), with substantial heterogeneity in prognosis [2]. DCM frequently represents a common terminal phenotype of diverse cardiomyopathies, and similar pathological features are observed in the end stages of hypertrophic, restrictive, and arrhythmogenic cardiomyopathies, further complicating diagnosis and therapeutic management [3]. Epidemiological data suggest a prevalence of approximately 1 in 250 in the general population [4], with a five-year post-diagnosis mortality rate of up to 20% [5]. As a leading cause of heart failure and cardiac transplantation, DCM imposes a significant healthcare burden [6,7].

The etiology of DCM is diverse and can be broadly categorized into genetic and acquired factors. Acquired triggers include viral infections, immune dysregulation, exposure to toxic substances (e.g., alcohol and chemotherapeutic agents), and metabolic disturbances [8]. Genetic variation also plays a major role, encompassing monogenic, oligogenic, and polygenic contributions that are frequently modulated by gene–environment interactions [9]. This complexity contributes to incomplete penetrance, variable expressivity, and uncertain genotype–phenotype correlations. Advances in molecular genetics have revealed that many cases previously classified as “idiopathic” have a genetic basis. Currently, genetic testing identifies pathogenic variants in approximately 20–40% of familial DCM and 15–25% of sporadic cases [1,10]. Over 200 genes have been implicated, encoding proteins involved in sarcomere structure, cytoskeleton integrity, nuclear envelope function, RNA splicing regulation, and ion channel activity, reflecting the marked genetic heterogeneity of DCM [9,11]. 

Increasing evidence supports a gene–environment “two hits” model in which inherited susceptibility interacts with environmental stressors to precipitate clinical disease, thereby contributing to phenotypic variability among carriers of the same mutation [3]. This concept has deepened mechanistic insights and informed updates in clinical management. With the rapid development of multi-omics technologies and advanced cardiac imaging, etiological diagnostic yield has improved substantially, enabling risk-stratified patient management and laying the foundation for targeted and precision interventions [12]. This review aims to systematically summarize the genetic underpinnings of DCM, delineate associated pathogenic mechanisms, and highlight recent advances in diagnosis and treatment, thereby providing a framework for individualized risk stratification and precision therapy.

## 2. Etiology and Pathogenic Mechanisms of DCM

**Figure 1:**
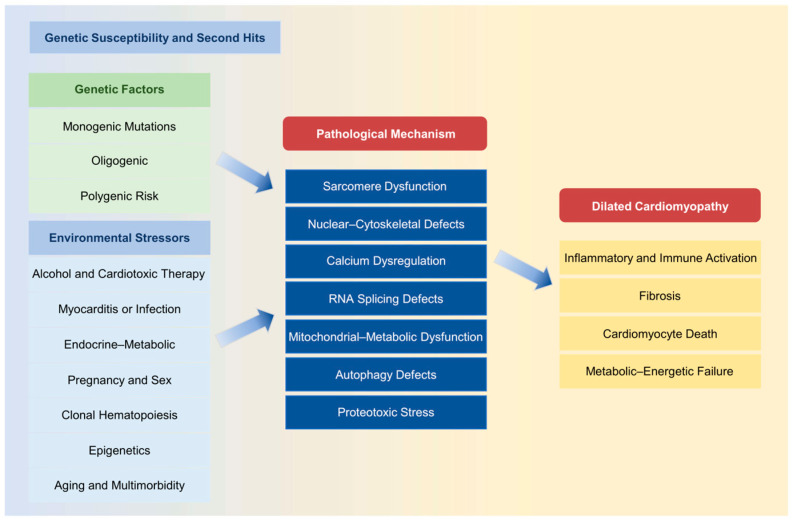
The etiology and mechanism of DCM. Schematic overview of the multilevel processes underlying DCM development and progression. Genetic susceptibility may arise from monogenic mutations, oligogenic inheritance, or polygenic risk. In predisposed individuals, environmental and acquired stressors, including alcohol exposure, cardiotoxic therapy, myocarditis or viral infection, endocrine–metabolic disorders, pregnancy, clonal hematopoiesis, epigenetic alterations, and aging-associated multimorbidity, act as modifiers or “second hits”. These influences converge on key intracellular pathways, such as sarcomere dysfunction, nuclear–cytoskeletal defects, calcium dysregulation, RNA splicing abnormalities, mitochondrial–metabolic impairment, autophagy disruption, and proteotoxic stress. Despite etiological heterogeneity, these mechanisms ultimately activate shared downstream processes, immune activation, fibrosis, cardiomyocyte death, and metabolic–energetic failure, driving adverse remodeling and clinical heart failure. DCM: Dilated cardiomyopathy.

As illustrated in Figure 1, DCM arises from interactions between diverse genetic architectures and environmental or acquired stressors that converge on shared molecular pathways within cardiomyocytes, ultimately driving maladaptive ventricular remodeling and heart failure progression.

### 2.1. Genetic Features

DCM exhibits autosomal dominant, recessive, X-linked, and mitochondrial inheritance patterns [13]. Autosomal dominant transmission is most prevalent, accounting for the majority of genetic DCM cases, though often with incomplete penetrance. 

The genetic architecture spans a continuum of pathogenicity [9]: (1) Monogenic: This type accounts for about 25–40% of familial DCM and 10–20% of sporadic cases [14], often driven by rare, high-impact variants in sarcomeric, cytoskeletal, or nuclear envelope genes, predominantly inherited in an autosomal dominant manner. (2) Oligogenic: Approximately 20–30% of cases may harbor 2–5 low or moderate effect variants affecting convergent pathways, whose combinatorial effects increase penetrance and phenotypic severity. (3) Polygenic: Numerous common variants of small effect, quantified using polygenic risk scores, cumulatively confer susceptibility and may interact with environmental triggers once a pathogenic threshold is exceeded.

### 2.2. Core Monogenic Pathogenic Mechanisms

To date, many genes have been implicated in DCM, although only a subset demonstrate robust gene–disease associations supported by genetic and experimental evidence [9]. Two independent studies applied semi-quantitative methods and case–control designs to systematically evaluate 51 and 56 DCM-associated genes, respectively, integrating clinical genetic and functional data [15,16]. In these analyses, 19 and 12 genes showed definitive associations with DCM and are therefore prioritized in clinical genetic testing. These genes encode proteins essential for cardiomyocyte structure, contractility, mechanotransduction, calcium handling, nuclear integrity, and RNA splicing, highlighting the marked molecular heterogeneity of this disorder (Table 1).

At the molecular level, pathogenic variants disrupt cardiac function through three principal mechanisms: haploinsufficiency, dominant-negative effects, and gain-of-function abnormalities [17]. These defects converge on shared pathways that impair myocardial contractility and promote maladaptive remodeling.

#### 2.2.1. Impaired Sarcomere Contractility

Sarcomeric dysfunction is a central pathogenic mechanism. Variants in genes encoding sarcomeric proteins directly impair force generation and energy efficiency, constituting the most direct molecular basis of DCM. Among these, truncating variants in *TTN* (TTNtvs) are the most common genetic cause, accounting for approximately 25% of familial cases and 18% of sporadic cases [18]. Penetrance of TTNtvs is age- and sex-dependent, with older male carriers showing more pronounced left ventricular dysfunction [19,20]. Moreover, pathogenicity also correlates with splice-isoform expression, as variants located in constitutively expressed cardiac exons are generally more deleterious and associated with worse prognosis [21]. Compared with other genetic forms of DCM, TTNtvs carriers often exhibit milder hypertrophy [22], better left ventricular reverse remodeling [23], and significantly increased risks of atrial and ventricular arrhythmias [24]. 

Pathogenic variants in *MYH7*, although more commonly associated with hypertrophic cardiomyopathy (HCM), also account for 3–4% of DCM cases [2]. In DCM, *MYH7* mutations impair contractility by reducing actin-activated ATPase activity and filament sliding velocity [25]. Other sarcomeric genes, including *MYBPC3*, *TNNT2*, *TNNI3*, *TNNC1*, *ACTC1*, and *TPM1*, similarly disrupt sarcomere stability and calcium sensitivity [26]. Collectively, these abnormalities initiate compensatory neurohormonal activation and metabolic stress, ultimately promoting ventricular dilation and systolic failure. 

#### 2.2.2. Disruption of Nuclear, Cytoskeletal, and Intercellular Connectivity

Cardiomyocyte integrity depends on coordinated interactions among the cytoskeleton, nuclear envelope, and intercellular junctions. Variants affecting these structures impair mechanotransduction and increase susceptibility to mechanically induced injury.

*LMNA* is the second most common DCM gene, accounting for approximately 10% of cases [27]. Pathogenic mechanisms include reduced nuclear mechanical stability and altered chromatin organization, linking structural nuclear defects to electrical abnormalities and progressive contractile dysfunction [28]. *LMNA*-associated DCM typically exhibits early onset, rapid progression, conduction disease, malignant arrhythmias [29], high risk of SCD, and poor left ventricular reverse remodeling [23].

Truncating variants in *FLNC* disrupt cytoskeletal structure and Z-disc integrity, impairing force transmission and promoting ventricular remodeling and arrhythmogenesis [30,31]. Similarly, mutations in desmosomal and intermediate filament genes such as *DSP* and *DES*, traditionally associated with arrhythmogenic cardiomyopathy, are increasingly recognized as causes of left-dominant DCM phenotypes [32]. 

#### 2.2.3. Calcium Homeostasis Imbalance

Disruption of intracellular calcium homeostasis is a key mechanism linking diverse genetic defects to impaired excitation–contraction coupling. Variants affecting calcium channels, pumps, and regulatory proteins reduce sarcoplasmic reticulum (SR) calcium reuptake or promote abnormal calcium leak, leading to diminished contractile reserve and increased arrhythmic susceptibility [33]. 

Pathogenic mutations in *SERCA2*, *PLN*, and *RYR2* are closely associated with DCM [34,35]. Reduced SERCA2a (encoded by *SERCA2*) activity and relatively enhanced *PLN*-mediated inhibition impair SR calcium reuptake and myocardial relaxation. Aberrant *RYR2*-mediated leak causes uncontrolled calcium release, depleting SR stores and triggering mitochondrial dysfunction and apoptosis [36]. Mutations in *SCN5A* may indirectly exacerbate calcium overload through sustained sodium influx [37]. Together, these abnormalities establish a vicious cycle of contractile dysfunction, mitochondrial stress, and cardiomyocyte injury, providing a mechanistic rationale for therapies targeting excitation–contraction coupling in selected DCM subtypes [38]. 

#### 2.2.4. Abnormal RNA Splicing Regulation

Variants in *RBM20* account for 1–5% of familial DCM [39]. *RBM20* encodes an RNA-binding protein that regulates alternative splicing of multiple key cardiac genes, including *TTN*, *CAMK2D*, and *RYR2* [40]. Missense variants in *RBM20* disrupt normal splicing patterns, producing dysfunctional protein isoforms that impair myocardial structure and function, leading to arrhythmias and systolic dysfunction [41]. These findings highlight the critical role of post-transcriptional regulation in maintaining cardiac homeostasis and suggest that RNA splicing defects represent a convergent mechanism across genetic cardiomyopathies. Clinically, *RBM20*-related DCM is characterized by early-onset, rapid progression, and high arrhythmic risk [42].

#### 2.2.5. Mitochondrial Dysfunction and Metabolic Impairment

Given the high energetic demands of the myocardium, mitochondrial dysfunction is a critical contributor to DCM. Primary mutations affecting mitochondrial structure or function reduce ATP production and increase oxidative stress [43]. 

*TAZ* mutations cause Barth syndrome, characterized by mitochondrial dysfunction and DCM with high early mortality [44]. *TAZ* encodes a phospholipid acyltransferase essential for cardiolipin remodeling and mitochondrial membrane stability [45]. *TAZ* deficiency impairs mitophagy, oxidative phosphorylation, and redox balance [46]. Conversely, cardiac overexpression of COUP-TFII (encoded by *NR2F2*) suppresses genes involved in electron transport, oxidative stress defense, and mitochondrial dynamics, increasing reactive oxygen species, reducing oxygen consumption, and impairing glucose and fatty acid oxidation via PGC-1α (encoded by *PPARGC1A*) downregulation, collectively promoting DCM progression [47]. Additionally, DCM myocardium exhibits mitochondrial DNA (mtDNA) repair defects, aberrant fission, and increased mtDNA deletions, further compromising oxidative phosphorylation [48]. 

#### 2.2.6. Autophagy Dysregulation

Autophagy is essential for maintaining proteostasis and organelle quality control in cardiomyocytes. Mutations in genes such as *BAG3* disrupt chaperone-assisted autophagy, leading to accumulation of misfolded proteins, cytoskeletal disorganization, and apoptosis [49,50]. Both insufficient and excessive autophagic activity are detrimental, underscoring the need for tightly regulated proteostatic balance. Aggregation-prone mutant proteins may both result from and further impair protein quality control, establishing a self-perpetuating cycle of proteotoxic stress.

#### 2.2.7. Proteotoxic Stress and Aberrant Protein Aggregation

Beyond defective degradation pathways, several DCM-associated mutations directly promote aberrant protein aggregation, representing a convergent downstream mechanism across genetic subtypes. Variants in structural or chaperone-related proteins, including *DES*, *CRYAB*, *PLN*, *TTN*, and *FLNC*, may generate misfolded or unstable proteins with intrinsic aggregation propensity [38,51,52,53]. These toxic aggregates disrupt sarcomeric integrity, cytoskeletal architecture, and intracellular signaling, while sequestering molecular chaperones, impairing ubiquitin–proteasome activity, and inhibiting autophagic flux. The resulting bidirectional interaction between protein aggregation and defective quality control establishes a vicious cycle of proteotoxic stress, ultimately driving progressive myocardial remodeling and contractile dysfunction.

### 2.3. Cumulative Effects of Multiple Genetic Variants

#### 2.3.1. Oligogenic Inheritance

Beyond monogenic inheritance, oligogenic effects also contribute to DCM. Emerging evidence suggests that at least 20–30% of DCM cases may have an oligogenic basis in which multiple rare variants located at distinct, non-linked loci collectively determine the final disease phenotype [9]. According to this model, a single variant may be insufficient to cause disease in isolation; however, the cumulative burden of multiple variants with low-to-moderate effect sizes may exceed the threshold required for clinical manifestation.

A multicenter European DCM cohort study reported that more than 38% of patients carried compound or combined variants, with 12.8% harboring three or more variants [54]. Similarly, in a study of *LMNA*-related cardiomyopathy, 26% of probands exhibited multiple interacting variants contributing to disease expression [55]. This framework provides a plausible explanation for the relatively modest diagnostic yield observed in some familial DCM cases and suggests that a more complex genetic architecture, rather than a single high-penetrance pathogenic variant, may underlie disease development in a substantial proportion of patients.

#### 2.3.2. Polygenic Inheritance

Genome-wide association studies (GWAS) demonstrate that common variants also contribute to DCM susceptibility [56]. Multiple susceptibility loci have been identified across diverse populations. In European DCM cohorts and matched controls, significant associations have been reported at chromosome 3p25.1 (encompassing *SLC6A6*), 22q11.23 (including *SMARCB1*) [57], 10q26 (including *BAG3*), and 1p36.13 (including *HSPB7*) [58], as well as an immune- and inflammation-related gene-enriched region at 6p21 [59]. In populations of African ancestry, intronic variants in *CACNB4* have also been identified [60].

In 2024, a large-scale GWAS integrating 9365 DCM cases and 946,368 controls identified 70 genome-wide significant loci through comprehensive GWAS and multitrait analyses. These loci were robustly replicated in independent cohorts and mapped to 63 prioritized genes. Tissue, cell type, and pathway enrichment analyses highlighted cardiomyocytes and the contractile apparatus as central components of DCM pathogenesis, providing a rationale for applying polygenic risk scores to assess disease susceptibility and predict clinical progression [61].

Although individually non-pathogenic, these variants act as modifiers of disease susceptibility and severity. Their effects can be conceptualized within a “threshold model”, in which the polygenic background modulates the phenotypic trajectory driven by rare pathogenic variants, thereby explaining the marked variability in penetrance and expressivity among carriers of identical disease-causing mutations.

### 2.4. Non-Genetic and Gene–Environment Interacting Causes of DCM

The development and progression of DCM reflect dynamic interactions between genetic susceptibility and environmental or systemic stressors. These factors may precipitate disease onset or accelerate progression, consistent with the gene–environment “second hits” framework described above.

#### 2.4.1. Alcohol Consumption and Cancer Therapy-Related Cardiotoxicity 

Chronic excessive alcohol intake exerts direct cardiotoxic effects through oxidative stress, mitochondrial injury, impaired excitation–contraction coupling, and cardiomyocyte apoptosis [62,63]. In genetically predisposed individuals, alcohol exposure may unmask latent cardiomyopathy and accelerate ventricular remodeling. 

Cancer therapy-related cardiotoxicity represents another major acquired trigger. Anthracyclines such as doxorubicin induce dose-dependent myocardial injury via oxidative stress, mitochondrial damage, calcium dysregulation, and topoisomerase IIβ-mediated DNA injury, resulting in largely irreversible cardiomyopathy [64,65]. Cardiac dysfunction may occur during therapy or emerge years later, contributing to an increasing burden among cancer survivors [66]. Trastuzumab disrupts neuregulin-1/ErbB survival signaling and typically causes reversible systolic dysfunction [67], whereas immune checkpoint inhibitors including nivolumab, pembrolizumab, and ipilimumab may trigger immune-mediated myocarditis through loss of immune tolerance and T cell-driven myocardial inflammation [68]. Furthermore, cardiac involvement is also common in immunoglobulin light-chain (AL) amyloidosis, where extracellular amyloid deposition and circulating light-chain toxicity drive progressive restrictive or dilated phenotypes [69,70].

Carriers of TTNtvs exposed to excessive alcohol or anthracyclines exhibit more severe systolic dysfunction and higher rates of heart failure and arrhythmias, underscoring the clinical relevance of gene–environment interactions [71,72].

#### 2.4.2. Myocarditis and Infection-Driven Immune–Inflammatory Injury

Viral infections such as parvovirus B19, coxsackievirus B, and adenovirus account for a substantial proportion of myocarditis cases, and approximately 20–30% of patients progress to chronic DCM [73]. Early injury results from direct viral cytotoxicity, mitochondrial dysfunction, and calcium-handling abnormalities, whereas persistent viral genomes and sustained immune activation promote chronic inflammation, extracellular matrix remodeling, and fibrosis, ultimately leading to ventricular dilation and systolic dysfunction [74]. 

Myocarditis encompasses diverse inflammatory phenotypes, including lymphocytic, eosinophilic, granulomatous, and giant cell myocarditis, which differ in clinical features and prognoses [75]. Lymphocytic myocarditis, the most common form, generally presents with mild to moderate symptoms but can progress to chronic ventricular dysfunction in a subset of patients. Eosinophilic myocarditis is often linked to hypersensitivity reactions or systemic eosinophilic syndromes and may rapidly lead to severe heart failure if untreated. Granulomatous myocarditis, such as in cardiac sarcoidosis, is characterized by patchy myocardial infiltration and conduction system involvement, predisposing patients to arrhythmias and sudden cardiac death. Giant cell myocarditis is rare but highly aggressive, frequently resulting in fulminant heart failure and life-threatening arrhythmias without prompt intervention. Overall, fulminant or immune-mediated forms are particularly associated with rapid deterioration and elevated arrhythmic risk.

Infection-triggered autoimmunity further contributes through mechanisms such as molecular mimicry and epitope spreading [76]. Circulating autoantibodies against cardiac antigens, detected in up to 60% of DCM patients, may exert cytotoxic and functional effects that promote progressive dysfunction, although their precise pathogenic role remains incompletely defined [73,77,78].

#### 2.4.3. Endocrine and Metabolic Disorders

Endocrine and metabolic disturbances contribute to myocardial dysfunction and may modify the clinical expression of genetic DCM [79]. In diabetes mellitus, chronic hyperglycemia and insulin resistance induce glucotoxicity, lipotoxicity, microvascular injury, and mitochondrial impairment, leading to fibrosis and progressive systolic dysfunction even in the absence of coronary disease [80]. Thyroid hormones regulate cardiac contractility and gene expression. Hyperthyroidism may cause tachycardia-induced cardiomyopathy and high-output failure, whereas hypothyroidism is associated with reduced contractility, impaired relaxation, and ventricular dilation [81]. Catecholamine excess, primary aldosteronism, growth hormone excess, and hypercortisolism promote myocardial injury through calcium overload, oxidative stress, inflammation, and fibrosis [82,83,84]. Importantly, many of these conditions are partially reversible with disease-specific therapy and may function as modifiable “second hits”. 

#### 2.4.4. Pregnancy and Sex-Related Factors

Pregnancy imposes substantial hemodynamic and hormonal stress. Peripartum cardiomyopathy (PPCM) exemplifies the interaction between physiological stressors and genetic susceptibility, with TTNtvs enriched in affected patients [85,86]. Proposed mechanisms include angiogenic imbalance, oxidative stress, impaired vascular signaling, and cardiotoxic prolactin fragments [87].

Sex differences also influence disease phenotype. Male patients often exhibit more pronounced systolic dysfunction and arrhythmic burden [88], whereas female patients may demonstrate less structural remodeling but variable progression patterns [89].

#### 2.4.5. Clonal Hematopoiesis (CH)

CH has recently emerged as an important risk factor associated with DCM. During normal aging, hematopoietic stem cells progressively accumulate random somatic mutations [90]. While most mutations are neutral, those occurring in driver genes such as *DNMT3A*, *TET2*, and *ASXL1* may confer a proliferative advantage, leading to clonal expansion of mutated hematopoietic cells—a phenomenon termed somatic mutation-driven CH [91,92]. A clinically representative subtype, CH of indeterminate potential (CHIP), is defined by the presence of somatic mutations in hematologic malignancy-associated genes in individuals without overt hematologic abnormalities, with a variant allele frequency (VAF) ≥ 2% in peripheral blood or bone marrow [93]. CHIP has been increasingly linked to non-hematologic diseases. In the cardiovascular field, accumulating evidence demonstrates strong associations between CHIP and elevated risks of coronary artery disease, heart failure, and arrhythmias, independent of traditional cardiovascular risk factors [94,95,96].

Data regarding the prognostic impact of CH in DCM remain limited. The largest cohort study to date included 520 patients with DCM and applied a highly sensitive VAF threshold of 0.01% to detect small clones, identifying CH driver mutations in 109 individuals. CH independently predicted both cardiac and all-cause mortality, irrespective of age or clone size [97]. Notably, sizable clones (VAF > 2%) have been detected even in patients younger than 30 years at a frequency exceeding that observed in the general population [98]. Another study evaluating CH as a predictor of cardiogenic shock analyzed 686 patients, 65% of whom had nonischemic cardiomyopathy. CH mutations were more prevalent among patients with cardiogenic shock and were associated with reduced survival compared with ambulatory heart failure patients [99].

The mechanisms by which CH contributes to DCM pathogenesis remain incompletely understood. Current evidence suggests that CH driver mutations enhance inflammatory signaling, including inflammasome activation, thereby amplifying systemic and myocardial inflammation [100]. This inflammatory milieu may further promote CH clone expansion, forming a self-reinforcing loop [101]. Beyond inflammation-dependent mechanisms, CH may also directly promote myocardial fibrosis. Monocytes isolated from heart failure patients harboring *DNMT3A* mutations stimulated the release of heparin-binding epidermal growth factor-like growth factor, leading to cardiac fibroblast activation and fibrosis [102]. Similarly, *TET2*-mediated CH exacerbated myocardial fibrosis in murine models of heart failure with preserved ejection fraction [103]. 

Collectively, these findings support the concept that CH may function as “second hits”, acting independently or synergistically with environmental stressors such as chemotherapy or inflammation to trigger or accelerate DCM progression in genetically susceptible hearts. This observation further suggests shared pathophysiological mechanisms between cancer biology and cardiovascular disease [104].

#### 2.4.6. Epigenetic Modifications

Environmental exposures can regulate gene expression through epigenetic mechanisms, including DNA methylation, histone modifications, and non-coding RNAs. Distinct methylation signatures have been identified in DCM myocardium and peripheral blood, correlating with disease status and gene expression [105]. Dysregulated histone modifications, including acetylation, methylation, and phosphorylation, activate pathways related to hypertrophy, fibrosis, and apoptosis [106,107]. Cardiac-enriched microRNAs (MiRNAs), such as miR-1, miR-133, and miR-208, further modulate structural remodeling and electrical stability, and their altered expression is associated with disease progression [108,109].

#### 2.4.7. Aging and Multimorbidity

Aging increases myocardial vulnerability through accumulated comorbidities, chronic inflammation, mitochondrial dysfunction, and reduced regenerative capacity. Age-related processes, including clonal hematopoiesis and cumulative environmental exposures, may interact with genetic predisposition to trigger disease onset and accelerate progression, highlighting aging as an important modifier within the gene–environment framework [19].

### 2.5. Shared Downstream Pathophysiological Pathways

The convergence of diverse forms of DCM on shared downstream pathways integrates the effects of primary genetic defects and secondary modifiers, ultimately driving adverse remodeling and heart failure progression. 

#### 2.5.1. Inflammation and Immunity

Inflammation represents a central pathway linking genetic susceptibility, environmental stressors, and disease progression in DCM. Notably, inflammatory cell infiltration and upregulated cytokine expression are also commonly observed in genetic DCM, likely reflecting innate immune activation in response to cardiomyocyte injury and the release of danger-associated molecular patterns. Single-cell RNA sequencing analyses have revealed profound transcriptional reprogramming of immune cell populations, including macrophages and T cells, within DCM myocardium. The degree of immune cell infiltration correlates with fibrosis severity and clinical outcomes [110]. While moderate inflammation in early disease stages may facilitate clearance of necrotic cells and initiate tissue repair, chronic inflammation sustains cytokine and growth factor release, creating a microenvironment that promotes maladaptive remodeling.

#### 2.5.2. Myocardial Fibrosis

Cardiac fibroblasts are the principal source and regulators of the extracellular matrix (ECM), capable of sensing mechanical signals transmitted from cardiomyocytes and adjusting proliferation and collagen secretion accordingly. Chronic inflammation and aberrant mechanical signaling continuously activate fibroblasts, driving their differentiation into myofibroblasts and leading to excessive collagen deposition and interstitial fibrosis. 

In *LMNA*-related DCM mouse models, cardiac fibroblasts undergo adaptive proliferation and activation through mechanosensing pathways dependent on p38 MAPK, resulting in excessive ECM secretion and driving disease severity. Pharmacological inhibition of p38 signaling attenuates fibroblast expansion and activation, thereby delaying disease progression [111]. 

Myocardial fibrosis increases stiffness and reduces ventricular compliance. These changes impair contractility and disrupt electrical conduction, providing a structural substrate for arrhythmias and progressive heart failure [112]. 

#### 2.5.3. Cardiomyocyte Death

Cardiomyocyte loss and replacement represent key processes in ventricular dilation and systolic failure. Increased expression of pro-apoptotic factors has been documented in DCM, leading to progressive depletion of functional cardiomyocytes [113]. Beyond classical apoptosis, emerging evidence suggests roles for pyroptosis and ferroptosis. Activation of the NLRP3 inflammasome and caspase-1 has been observed in myocardial tissue from DCM patients and animal models, inducing cardiomyocyte pyroptosis [114]. Bioinformatics analyses have identified upregulation of ferroptosis-related genes such as *STAT3* in DCM, suggesting potential involvement of this pathway [115]. Although direct evidence remains limited, the central role of oxidative stress in DCM supports ferroptosis as a plausible contributor to cardiomyocyte loss.

Although the relative contribution of individual death pathways in human DCM remains incompletely defined, cumulative cardiomyocyte loss represents a final common pathway that accelerates structural remodeling and functional decline, regardless of the initiating genetic defect.

#### 2.5.4. Metabolic Remodeling and Energetic Failure

Metabolic remodeling is a consistent feature of DCM and heart failure, characterized by impaired oxidative phosphorylation, altered substrate utilization, and reduced metabolic flexibility [116]. Although initially adaptive—since glucose metabolism generates ATP with lower oxygen consumption—chronic reliance on glycolysis leads to lactate accumulation and intracellular acidosis, impairing cellular function [117]. 

Primary mitochondrial dysfunction, as well as secondary metabolic impairment resulting from sarcomeric inefficiency or calcium dysregulation, leads to energy deficiency and increased oxidative stress [118]. In TTNtvs rat models, cardiomyocytes exhibit marked metabolic defects, including mitochondrial dysfunction, increased reactive oxygen species production, and altered tricarboxylic acid cycle intermediates [119]. These changes not only reduce energy supply but also generate toxic byproducts that intensify intracellular acidosis, reinforcing a vicious cycle. Reduced *LMNA* expression shifts cardiac metabolism toward glycolysis and away from medium- and long-chain fatty acid oxidation, potentially activating mTORC1 signaling, promoting ineffective protein synthesis and autophagy, and further aggravating myocardial injury and systolic dysfunction [120]. Moreover, inter-organelle communication is essential for cardiomyocyte homeostasis. Recent studies indicate that miR-16-5p mediated endoplasmic reticulum stress promotes mitochondrial dysfunction in human cardiomyocytes [121]. 

Energetic failure exacerbates contractile dysfunction and sensitizes cardiomyocytes to injury, reinforcing a vicious cycle of metabolic stress and myocardial damage. These observations provide a rationale for emerging therapies aimed at improving myocardial energetics and mitochondrial function in selected patient subsets.

## 3. Clinical Evaluation and Diagnostic Approach

### 3.1. Comprehensive Clinical Assessment

The diagnosis of DCM requires a systematic and comprehensive clinical evaluation. A detailed medical history should assess the mode of symptom onset, rate of progression, prior arrhythmic events, pregnancy history, exposure to cardiotoxic agents, and features suggestive of infectious or autoimmune disease. A thorough family history is essential, particularly regarding early-onset heart failure or SCD. Integration of these clinical features provides the framework for subsequent investigations, including electrophysiological testing, imaging, laboratory studies, and genetic evaluation, thereby enabling an etiology-oriented diagnostic strategy.

### 3.2. Electrophysiological Assessment

A standard 12-lead electrocardiogram (ECG) and ambulatory ECG monitoring are fundamental tools in the evaluation of DCM. Abnormal ECG findings are present in most patients and commonly include QRS or PR prolongation, left bundle branch block, and nonspecific ST–T wave abnormalities. Although traditionally considered nonspecific, emerging genotype–phenotype studies indicate that certain electrocardiographic patterns may suggest underlying genetic subtypes. For example, *LMNA*-related DCM frequently manifests with sinus bradycardia or atrioventricular conduction block, whereas *DSP*- or *FLNC*-associated disease may present with low QRS voltage or lateral T-wave inversion [122].

In patients at risk for arrhythmias, ambulatory ECG monitoring facilitates detection of frequent premature ventricular complexes or nonsustained ventricular tachycardia. Microvolt T-wave alternans has been explored as a non-invasive tool for SCD risk stratification [123], and its relatively high negative predictive value may help identify low-risk individuals. Signal-averaged ECG and novel surface ECG-derived markers of repolarization instability, such as the regional restitution instability index and peak ECG restitution slope, have demonstrated incremental value in research settings [124,125]. Compared with conventional tools, these parameters may offer improved predictive performance and assist in selecting patients most likely to benefit from implantable cardioverter-defibrillator (ICD) therapy, although routine clinical application requires further validation.

### 3.3. Laboratory and Biomarker Assessment

#### 3.3.1. Etiology-Oriented Laboratory Screening

Initial laboratory investigations aim to identify potentially reversible or secondary causes of myocardial dysfunction. Measurement of thyroid-stimulating hormone and thyroid hormone levels is recommended as thyroid dysfunction may lead to reversible cardiomyopathy. Disorders of iron metabolism, such as hereditary hemochromatosis or iron overload, should be evaluated using serum ferritin and transferrin saturation. In patients with clinical features suggestive of systemic autoimmune disease, testing for antinuclear antibodies and related autoantibody panels may be appropriate. Depending on the clinical context, viral serologies, metabolic profiling, and additional endocrine assessments may also be considered. Such etiology-directed screening facilitates the identification of reversible contributors and may significantly influence therapeutic strategies.

#### 3.3.2. Biomarkers of Myocardial Injury and Remodeling

Natriuretic peptides and cardiac troponins remain the most widely used biomarkers in clinical practice. Natriuretic peptides reflect myocardial wall stress, whereas troponins indicate ongoing myocardial injury; both are independently associated with all-cause mortality [126]. Biomarkers involved in myocardial remodeling and inflammatory pathways, including soluble ST2, galectin-3, and tissue inhibitors of metalloproteinases, have also demonstrated prognostic value in mixed-etiology DCM populations [127,128,129]. 

Given the multifactorial pathophysiology of DCM, reliance on a single biomarker may not adequately capture disease activity. Multimarker strategies that integrate signals from distinct pathways appear to improve risk prediction. In a prospective cohort of patients with chronic heart failure, approximately half of whom had non-ischemic DCM, incorporation of NT-proBNP, high-sensitivity troponin, and ST2 into a risk model significantly improved discrimination compared with any single biomarker alone [130]. These findings suggest that complementary biological signals may enable more refined and individualized risk stratification.

### 3.4. Imaging Assessment

Transthoracic echocardiography is the first-line imaging modality for diagnosing DCM, confirming left ventricular dilatation and systolic dysfunction while excluding valvular or congenital heart disease. Beyond left ventricular ejection fraction, advanced parameters such as global longitudinal strain and mechanical dispersion can detect early myocardial dysfunction and may provide superior predictive value for ventricular arrhythmias [131,132]. These measures should, however, be interpreted within the broader clinical context.

Cardiac magnetic resonance (CMR) is recommended when diagnostic uncertainty persists or when detailed tissue characterization is required. CMR provides precise quantification of ventricular volumes and function and identifies focal myocardial fibrosis using late gadolinium enhancement (LGE). The presence of LGE is associated with increased risks of ventricular arrhythmias and adverse cardiovascular outcomes and serves as an important marker for risk stratification [133]. Parametric techniques, including T1 mapping, extracellular volume quantification, and T2 mapping, enable assessment of diffuse fibrosis and myocardial edema and may provide incremental information even in the absence of detectable LGE [134].

Although not routinely indicated in all patients, positron emission tomography–computed tomography may be useful when inflammatory or infiltrative etiologies are suspected, as it allows assessment of myocardial metabolic activity and inflammation and may assist in guiding biopsy [135].

### 3.5. Endomyocardial Biopsy

Endomyocardial biopsy (EMB) should be considered in patients with unexplained heart failure of recent onset, particularly when accompanied by hemodynamic instability, malignant ventricular arrhythmias, or lack of response to standard therapy [136]. EMB enables histopathological evaluation, immunohistochemical characterization, and viral genome analysis, facilitating the diagnosis of inflammatory or infiltrative cardiomyopathies and guiding targeted therapies, including immunosuppression or antiviral therapy. Despite its diagnostic value in selected clinical scenarios, the invasive nature of EMB, potential sampling error, and procedural risks limit its routine use. Accordingly, EMB should be performed in carefully selected patients at experienced centers.

### 3.6. Genetic Evaluation

Genetic testing has become an integral component of etiological assessment in DCM. Identification of a pathogenic variant not only confirms the underlying cause but also informs family screening and risk stratification. Indications include early-onset disease (typically < 50 years), a family history of DCM or premature SCD, conduction system abnormalities, extracardiac manifestations, or sporadic cases with unexplained etiology [1]. Importantly, even in the presence of acquired triggers, such as excessive alcohol consumption, anthracycline-induced cardiotoxicity, or PPCM, underlying genetic susceptibility should be considered, as pathogenic variants may be enriched in these subgroups.

Beyond etiological clarification, genetic findings also carry prognostic implications. In a multicenter Spanish cohort of 1005 DCM probands, pathogenic variant carriers showed significantly higher rates of major adverse cardiac events, end-stage heart failure, and malignant ventricular arrhythmias than non-carriers [137]. Distinct genes are associated with specific clinical trajectories and arrhythmic risk profiles. Consequently, genotype-informed classification, integrated with clinical and imaging data, may enhance personalized risk stratification and therapeutic decision-making [138].

## 4. Treatment

**Figure 2:**
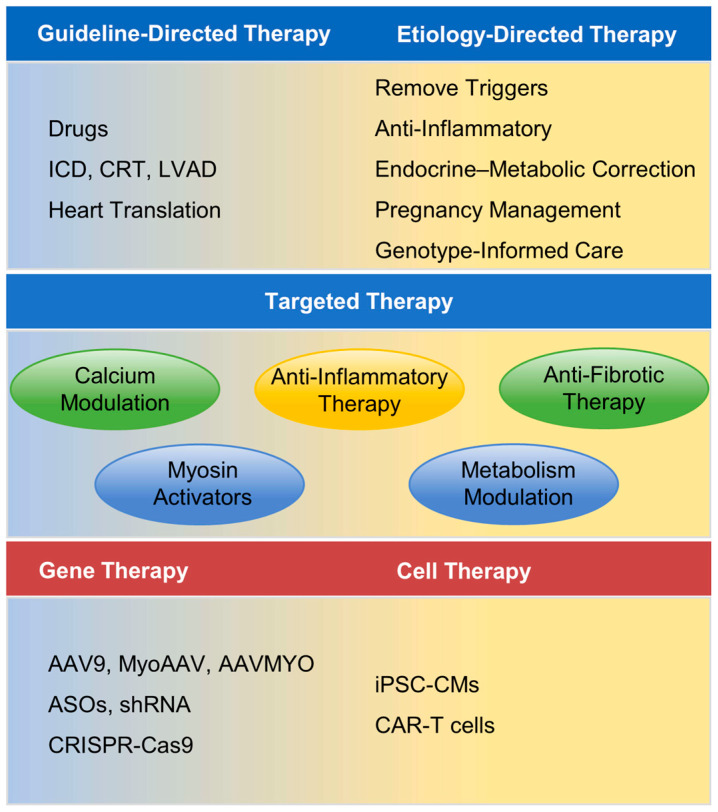
Mechanism- and etiology-informed therapeutic strategies for DCM. Conceptual framework integrating guideline-directed therapy, etiology-directed management, targeted pathway modulation, and gene- and cell-based interventions in DCM. In addition to standard pharmacologic and device therapies (including ICD, CRT, LVAD, and transplantation), etiology-directed approaches, such as trigger removal, anti-inflammatory treatment, endocrine–metabolic correction, pregnancy management, and genotype-informed care address disease-specific drivers. Advances in genetic and molecular characterization enable stratification by predominant pathogenic mechanisms, supporting targeted therapies aimed at calcium handling, sarcomeric or myosin dysfunction, inflammation, fibrosis, and metabolic remodeling. Gene- and cell-based strategies represent emerging adjunctive approaches that may complement established heart failure management in selected patients. DCM: Dilated cardiomyopathy; ICD: Implantable cardioverter-defibrillator; CRT: Cardiac resynchronization therapy; LVAD: Left ventricular assist device; AAV: Adeno-associated virus; ASOs: Antisense oligonucleotides; iPSC-CMs: induced pluripotent stem cell-derived cardiomyocytes; CAR-T: Chimeric antigen receptor T cell therapy.

Figure 2 summarizes current and emerging therapeutic strategies for DCM, highlighting the transition from conventional guideline-directed therapy toward mechanism-based precision medicine and gene- or cell-based interventions (Table 2). 

### 4.1. Conventional Therapy

Management of DCM primarily relies on pharmacological treatment targeting heart failure, arrhythmias, and thromboembolic complications. All patients should receive guideline-directed medical therapy (GDMT) to reduce mortality and delay disease progression. Contemporary “quadruple therapy” includes renin–angiotensin system inhibitors, β-blockers, mineralocorticoid receptor antagonists, and sodium–glucose cotransporter 2 inhibitors, with adjunctive use of diuretics, digoxin, or ivabradine as clinically indicated.

For patients with inadequate response to medical therapy or increased risk of malignant arrhythmias, device-based interventions should be considered. These include ICD for SCD prevention, cardiac resynchronization therapy to improve ventricular synchrony and function, and left ventricular assist devices for circulatory support in end-stage heart failure. When all other therapies fail, heart transplantation remains the only potentially curative option, although its use is limited by donor availability and post-transplant complications. 

### 4.2. Etiology-Directed and Genotype-Guided Therapy

Although GDMT remains the foundation of treatment, recognition of etiological heterogeneity has important therapeutic implications. Identification of disease-specific drivers may attenuate adverse remodeling, reduce arrhythmic risk, and improve clinical outcomes.

#### 4.2.1. Genetic DCM

Genetic DCM comprises a heterogeneous spectrum of pathogenic variants that significantly influence disease trajectory and arrhythmic susceptibility. Variants in high-risk genes such as *LMNA*, *FLNC*, *PLN*, *RBM20*, *DSP*, and *DES* are strongly associated with malignant ventricular arrhythmias, conduction system disease, and SCD. In affected individuals, risk-adapted early ICD implantation may be considered even when left ventricular ejection fraction exceeds the conventional 35% threshold, particularly in the presence of additional markers such as non-sustained ventricular tachycardia or extensive fibrosis on CMR imaging [138].

In contrast, carriers of TTNtvs often demonstrate favorable reverse remodeling under GDMT, supporting careful longitudinal reassessment before prophylactic device escalation. These observations highlight the value of genotype-informed risk stratification and therapeutic decision-making.

#### 4.2.2. Cardio-Oncology-Associated Cardiomyopathy and Infiltrative Disease

Cancer therapy-related cardiotoxicity has become an increasingly important cause of secondary DCM. Anthracycline-induced cardiomyopathy is typically dose-dependent and often irreversible, necessitating cumulative dose limitation, serial cardiac surveillance, and, in selected high-risk individuals, cardioprotective strategies. Early initiation of GDMT is recommended once left ventricular dysfunction is detected. 

By contrast, trastuzumab-associated cardiac dysfunction is generally not dose-dependent and is often reversible. Early detection, temporary treatment interruption, and optimization of heart failure therapy may allow recovery of ventricular function. Immune checkpoint inhibitors may precipitate fulminant immune-mediated myocarditis with high mortality; management requires immediate discontinuation of the offending agent and prompt initiation of high-dose corticosteroid therapy, with escalation to additional immunosuppression when necessary. 

Cardiac amyloidosis, particularly AL and transthyretin subtypes, may present with ventricular dysfunction mimicking DCM. AL amyloidosis requires urgent hematologic therapy targeting the underlying plasma cell clone, whereas transthyretin amyloidosis may be treated with disease-modifying therapies aimed at stabilizing or silencing transthyretin. Recognition of infiltrative cardiomyopathy is therefore critical, as management differs fundamentally from primary DCM.

#### 4.2.3. Inflammatory Cardiomyopathy and Myocarditis

DCM secondary to inflammatory myocardial injury requires subtype-specific therapeutic strategies [75]. Lymphocytic myocarditis, commonly related to viral infection or autoimmune mechanisms, may benefit from antiviral therapy or immunosuppression in carefully selected patients, particularly when biopsy confirms virus-negative inflammatory cardiomyopathy. 

Eosinophilic myocarditis is frequently associated with hypersensitivity reactions or systemic inflammatory disorders and is typically treated with corticosteroids alongside management of the underlying condition. 

Granulomatous myocarditis, including cardiac sarcoidosis, and giant cell myocarditis are characterized by aggressive inflammation, conduction abnormalities, and high arrhythmic burden. Early initiation of immunosuppressive therapy, often combining corticosteroids with additional immunomodulatory agents, may substantially influence prognosis.

#### 4.2.4. Endocrine and Metabolic Cardiomyopathies

Endocrine disorders represent important and potentially reversible causes of DCM. Diabetic cardiomyopathy is driven by metabolic dysregulation, oxidative stress, and myocardial fibrosis. Optimal glycemic control and contemporary cardioprotective therapies may attenuate adverse remodeling [139]. Correction of thyroid hormone imbalance frequently results in partial or complete recovery of ventricular dysfunction. 

In pheochromocytoma, excessive catecholamine exposure may cause reversible or persistent systolic dysfunction, and surgical resection following appropriate α-adrenergic blockade often restores cardiac function. Acromegaly, characterized by chronic growth hormone and insulin-like growth factor-1 excess, may progress to DCM in advanced stages, and effective hormonal control can stabilize or improve myocardial performance. Collectively, these conditions underscore the importance of identifying reversible endocrine drivers, as targeted therapy may substantially modify disease trajectory.

#### 4.2.5. PPCM

In PPCM, standard heart failure therapy remains the foundation of treatment. Bromocriptine, a prolactin secretion inhibitor, has shown potential benefit in selected patients when combined with anticoagulation and GDMT [140]. Although recovery of ventricular function is common, relapse may occur in subsequent pregnancies, particularly among women with incomplete recovery, highlighting the need for careful preconception counseling and long-term follow-up.

### 4.3. Emerging Targeted Therapies

#### 4.3.1. Targeting the Myofilament and Contractile Function

Because impaired sarcomeric contractility represents a central pathogenic mechanism in DCM, enhancing myocardial contractile performance is a rational therapeutic strategy. Omecamtiv mecarbil (OM) is a selective cardiac myosin activator that augments systolic function by prolonging actin–myosin cross-bridge attachment time without increasing intracellular calcium or myocardial energy consumption [141]. Importantly, OM demonstrates therapeutic potential not only in patients harboring sarcomeric variants such as *TTN* and *MYH7*, but also in those with non-sarcomeric etiologies. In the phase II COSMIC-HF trial, OM significantly improved cardiac systolic function in patients with chronic heart failure and reduced ejection fraction [142]. Subsequently, the large-scale GALACTIC-HF trial, enrolling over 8000 patients, showed that OM reduced NT-proBNP levels, lowered the risk of heart failure events, improved clinical symptoms, and exhibited a favorable safety profile [143].

Danicamtiv is a next-generation myosin activator designed to provide greater contractile enhancement while minimizing adverse effects on diastolic function and promoting reverse remodeling [144]. A phase II clinical trial showed that danicamtiv was well tolerated and significantly improved left ventricular and left atrial echocardiographic parameters compared with placebo [145]. In another phase II study, danicamtiv enhanced myosin function and myofilament force generation, with particularly pronounced benefits in patients carrying *MYH7* or *TTN* variants [146]. Collectively, these findings highlight myofilament activation as a promising direction for etiology-informed precision therapy in DCM. 

#### 4.3.2. Targeting Ion Channels and Calcium Homeostasis

Disordered intracellular calcium handling is a key contributor to impaired excitation–contraction coupling in DCM. Pharmacological strategies that enhance myofilament calcium sensitivity may therefore improve contractile performance and mitigate cardiomyocyte dysfunction. Voltage-dependent anion channel 2 (VDAC2) is a mitochondrial outer membrane pore protein that regulates cytosolic–mitochondrial calcium signaling. Loss of VDAC2 markedly disrupts excitation–contraction coupling. In pressure overload-induced heart failure mouse models, activation of VDAC2 by efsevin improved cardiac contractility, identifying VDAC2 as a potential therapeutic target [147]. 

The gain-of-function *SCN5A* R222Q variant is associated with severe arrhythmogenic DCM and has been identified in multiple families. Affected patients often respond poorly to standard heart failure therapy; however, sodium channel-blocking agents can markedly improve systolic function and reduce arrhythmic burden [148]. Additionally, single systemic administration of SERCA2a gene therapy using adeno-associated virus (AAV) serotype 9 vectors in Duchenne muscular dystrophy mouse models produced sustained effects for up to 18 months, improving calcium cycling, enhancing muscle function, and preventing the onset and progression of lethal cardiomyopathy [149].

#### 4.3.3. Targeting Inflammatory and Immune Pathways

Given the critical role of inflammation in specific DCM subtypes, modulation of inflammatory signaling has emerged as a promising therapeutic strategy. IL-1 inhibition has been reported to improve cardiac function in patients with idiopathic DCM [150]. Therapeutic targeting of the IL-17 pathway is also under investigation, aiming to interrupt mechanisms that drive progression from myocarditis to DCM [151]. Moreover, strategies directed at virus-associated immune pathways, such as the fractalkine/CX3CR1 axis [152], or suppression viral replication using antimicrobial peptides including CRAMP and LL-37 [153], may offer additional therapeutic avenues for virus-related DCM.

Inhibition of inflammation associated with CH, including pathways involving IL-1β, IL-6, TNF, NLRP3, or JAK signaling, has been shown to mitigate CH-driven inflammatory burden. In heart failure mouse models, *TET2* loss–associated CH promotes adverse cardiac remodeling through IL-1β–mediated mechanisms. Consequently, patients with *TET2*-driven CH may particularly benefit from IL-1β/NLRP3 inflammasome inhibition [100]. In high-risk cardiovascular patients harboring *TET2* mutations, treatment with the IL-1β-neutralizing antibody canakinumab reduced major adverse cardiovascular events by 62%, compared with only 7% in CH-negative controls [154]. Colchicine, a broad-spectrum anti-inflammatory agent that inhibits microtubule polymerization and partially suppresses NLRP3 inflammasome activation, has demonstrated efficacy across multiple cardiovascular conditions [155]. In *TET2* mutant CH mouse models, colchicine suppressed IL-1β elevation and prevented accelerated atherosclerosis [156]. Similarly, a clinical trial in patients with chronic coronary artery disease showed that low-dose colchicine limited expansion of *TET2* CH clones and reduced cardiovascular risk [157].

#### 4.3.4. Targeting Fibrosis

Myocardial fibrosis represents a shared downstream pathway in DCM progression, making antifibrotic therapy an attractive target. In addition to conventional agents such as angiotensin-converting enzyme inhibitors and mineralocorticoid receptor antagonists, which exert established antifibrotic effects, more specific antifibrotic therapies are under development. Antibodies or small-molecule inhibitors targeting the TGF-β signaling pathway have demonstrated antifibrotic efficacy and improved cardiac function in preclinical models [158]. 

ARRY-371797, a p38 MAPK inhibitor targeting cardiac fibroblast activation, prevented further left ventricular dilation and functional decline in *LMNA* mutant mouse models [111]. In a phase II trial, ARRY-371797 reduced NT-proBNP levels and improved heart failure symptoms [159]. However, the subsequent phase III trial was terminated early due to insufficient efficacy [160], likely reflecting the complex biology of *LMNA*-related disease and heterogeneity in downstream responses. 

#### 4.3.5. Targeting Energy Metabolism

Disrupted myocardial energy metabolism is a hallmark of DCM, making metabolic modulation an important therapeutic strategy. Sodium–glucose cotransporter 2 inhibitors improve myocardial energetics, reduce oxidative stress and inflammation, and promote reverse remodeling [161]. Supplementation with metabolic intermediates, such as L-carnitine to enhance fatty acid oxidation, and agents including trimetazidine or perhexiline, which partially inhibit fatty acid oxidation while promoting glucose utilization, have also shown potential benefit [162,163,164].

An innovative study demonstrated that cardiac overexpression of phosphoglycerate dehydrogenase via gene therapy enhanced serine biosynthesis in DCM mouse models, improving cardiac function, reducing fibrosis, and prolonging survival. These findings provide new insight into therapeutic modulation of one-carbon metabolism in DCM [165].

### 4.4. Gene Therapy

Gene therapy aims to directly correct or compensate for pathogenic genetic defects, thereby interrupting disease progression at its source, and represents a major future direction for DCM treatment. 

#### 4.4.1. Gene Replacement Therapy

For haploinsufficient variants, delivery of a functional transgene represents the most advanced gene replacement strategy in clinical development. This approach preserves the patient’s endogenous DNA sequence and is associated with a favorable safety profile. 

For example, AAV9-mediated delivery of wild-type *NEXN* cDNA restored cardiac function and prolonged survival in loss-of-function *NEXN* mutant mouse models [166]. Similarly, AAV9-mediated *Mybpc3* delivery in neonatal mice with homozygous *Mybpc3* deficiency increased protein expression and suppressed cardiac remodeling for up to 30 weeks [167]. In *MYBPC3*-deficient HCM mouse models, TN-201, an optimized AAV9 vector incorporating a minimized promoter and cis-regulatory elements, efficiently delivered and expressed the target gene. At clinically relevant doses, TN-201 reversed structural abnormalities, improved cardiac function, and extended survival [168]. In a phase I clinical trial, TN-201 increased myocardial *MYBPC3* expression, reduced key biomarkers of cardiac injury and left ventricular hypertrophy, and demonstrated overall favorable efficacy [169].

#### 4.4.2. Gene Silencing Therapy

For gain-of-function or dominant-negative variants, small interfering RNA, short hairpin RNA, or antisense oligonucleotides (ASOs) can be designed to selectively degrade mutant mRNA or block its translation, achieving allele-specific silencing and elimination of pathogenic proteins. A critical prerequisite is confirmation that a single functional allele is sufficient to meet physiological and stress-related protein demands, and that haploinsufficiency itself does not cause disease.

In *PLN* R14del mutant DCM mouse models, AAV-delivered ASOs targeting *PLN* mRNA prevented mutant protein aggregation and consequently attenuated ventricular dilation and dysfunction [170]. Allele-specific silencing using shRNA has also been proposed for *LMNA*-associated cardiomyopathy [171]. Additionally, allele-specific nuclease delivery systems may enable permanent inactivation of mutant DNA sequences [172].

Given the challenge of individually correcting thousands of cardiomyopathy-associated missense variants, selective gene silencing targeting shared single-nucleotide polymorphisms has emerged as a promising alternative. RNA interference directed at such mutation-associated polymorphisms suppressed HCM phenotypes for up to six months in mouse models, with as little as a 25% reduction in mutant transcripts sufficient to prevent disease onset [173]. This strategy circumvents the need for variant-specific therapies and supports the feasibility of low-dose, repeat treatment, provided that high allele specificity is maintained to avoid haploinsufficiency-related risks.

#### 4.4.3. Gene Editing

Direct correction of pathogenic variants at the genomic DNA level using gene-editing technologies such as CRISPR–Cas9 represents the most definitive, yet technically challenging, therapeutic approach. The system employs guide RNA-directed Cas9 nuclease to introduce site-specific DNA cleavage, followed by non-homologous end joining or homology-directed repair to achieve gene disruption or precise correction. Feasibility has been demonstrated in DCM-related cellular and animal models. For instance, genome editing targeting TTNtvs in stem cell models increased titin protein levels and rescued *TTN*-associated functional defects [174]. Similarly, selective correction of the mutant allele while preserving wild-type *PLN* function improved cardiac performance in mice carrying the human *PLN* R14del variant [175]. 

Despite these advances, several challenges limit clinical translation, including low editing efficiency in cardiomyocytes, potential off-target effects, and immunogenicity of Cas9 proteins. To address these limitations, next-generation tools such as base editors and prime editors have been developed. Base editors enable precise single-nucleotide conversions (C-G to T-A or A-T to G-C) without inducing double-strand breaks [176], whereas prime editors combine Cas9 nickase with reverse transcriptase to mediate highly accurate insertions, deletions, or substitutions [177]. These platforms are particularly suitable for terminally differentiated cardiomyocytes because they do not rely on inefficient homology-directed repair and offer improved precision with reduced off-target risk.

A key advance facilitating *in vivo* cardiac gene editing is the development of myotropic AAV vectors, including MyoAAV and AAVMYO. Conventional serotypes such as AAV9 exhibit strong cardiac tropism but often require high doses, increasing off-target transduction and immunogenicity [178,179,180]. MyoAAV and AAVMYO were engineered through directed evolution and capsid modification to enhance cardiomyocyte specificity, enabling efficient delivery of gene-editing tools at lower doses while minimizing non-cardiac exposure [181,182]. These vectors have been successfully applied in preclinical studies. For example, MyoAAV 2A-mediated delivery of base editors targeting oxidation-sensitive methionine residues in CaMKIIδ improved cardiac function and reduced fibrosis after ischemia/reperfusion injury [183]. Similarly, AAVMYO combined with base editors repaired pathogenic *RBM20* mutations, restoring normal splicing and cardiac function in knock-in mouse models, with no detectable off-target editing [184]. Collectively, these findings highlight engineered myotropic AAVs as efficient and tissue-specific platforms for *in vivo* cardiac gene editing and support their potential clinical translation.

### 4.5. Cell Therapy

Cell-based therapies represent another rapidly evolving area in DCM research, with induced pluripotent stem cells (iPSCs) being the most widely used platform. iPSCs possess self-renewal capacity and multilineage differentiation potential and can be combined with CRISPR/Cas9-mediated gene editing to correct pathogenic variants in patient-derived cells. After differentiation into cardiomyocytes, corrected cells may be transplanted back into patients. These cells not only replenish damaged myocardium directly but also secrete exosomes that deliver cardioprotective molecules via paracrine mechanisms, thereby rescuing neighboring injured cells [185]. This strategy enables personalized therapy and provides a valuable platform for dissecting gene-specific disease mechanisms and variant pathogenicity. Nevertheless, challenges remain, including the risk of post-transplant arrhythmias, highlighting the need for improved safety strategies.

Beyond direct therapeutic application, iPSC-derived cardiomyocytes (iPSC-CMs) also serve as robust platforms for gene-directed therapy development and drug screening. Patient-derived iPSC-CMs carrying pathogenic variants in genes such as *LMNA*, *DES*, *TNNT2*, *PLN*, *RBM20*, *TTN*, and *BAG3* recapitulate disease phenotypes *in vitro*, enabling mechanistic studies, gene correction, and pharmacological screening to ameliorate pathological features [186,187,188].

In addition, chimeric antigen receptor T cell (CAR-T) therapy has opened new therapeutic avenues. CAR-T cells can be engineered to selectively recognize pathological cells, including CH clones, enabling targeted immune-mediated clearance [189]. CAR-T cells targeting fibroblast activation protein have been shown to eliminate activated cardiac fibroblasts, attenuate myocardial fibrosis, and improve cardiac function [190]. Integration of gene editing with cellular immunotherapy therefore represents a promising and innovative therapeutic paradigm for DCM and other cardiovascular diseases.

In summary, the therapeutic landscape of DCM is progressively transitioning from symptom-based management toward mechanism-oriented and genotype-informed precision care. The integration of conventional heart failure therapy with etiology-specific interventions, targeted molecular modulation, gene correction strategies, and regenerative approaches reflects a paradigm shift in disease management. As genetic screening, multimodal imaging, and biomarker profiling become increasingly embedded in clinical practice, individualized therapeutic algorithms are likely to replace uniform treatment pathways. Such a framework holds the potential to optimize risk stratification, prevent adverse remodeling, and ultimately improve long-term outcomes in this heterogeneous cardiomyopathy.

## 5. Summary and Perspectives

DCM is characterized by profound etiological and clinical heterogeneity, rendering its diagnosis and management inherently complex. Genetic factors play a central role in both disease initiation and progression. Current understanding of the genetic architecture has evolved beyond classical Mendelian inheritance to encompass oligogenic effects, polygenic risk, gene–environment interactions, and epigenetic regulation. At the mechanistic level, impaired contractility, defective mechanotransduction, calcium-handling abnormalities, metabolic dysregulation, inflammation, and fibrosis are closely interconnected and collectively drive disease pathogenesis. Elucidation of these pathways has provided a critical foundation for identifying novel therapeutic targets. Nevertheless, despite substantial advances in DCM genetics, important challenges remain.

### 5.1. Uncertainty in Gene and Variant Pathogenicity

Genomic technologies have identified numerous genes and variants associated with DCM; however, the lack of robust functional validation frameworks limits precise delineation of how specific variants perturb myocardial structure and function. Consequently, causal evidence linking many candidate genes and variants to DCM remains insufficient, hindering translation from gene discovery to mechanistic insight and complicating clinical interpretation. Standardized criteria for variant classification, phenotype definition, and analytical pipelines are therefore urgently needed to reduce inter-study variability and enhance clinical relevance.

Large-scale, systematic assessment of gene and variant pathogenicity using high-throughput functional genomics and iPSC-CM platforms, together with continuous updates to public resources such as ClinVar and ClinGen, will facilitate more accurate variant interpretation and rational gene panel design. Moreover, a substantial proportion of patients still lack a definitive genetic diagnosis after testing. Identifying additional disease-causing genes through larger and more diverse cohorts remains essential to improve diagnostic yield and enable cascade screening in affected families. 

### 5.2. Incomplete Genotype–Phenotype Correlations

DCM exhibits marked locus and allelic heterogeneity. Variants within the same gene may produce divergent clinical phenotypes and trajectories, whereas mutations in distinct genes can converge on similar presentations. This complex and non-linear genotype–phenotype relationship represents a major barrier to precision medicine. Future studies should clarify how specific variants influence disease onset, progression, and prognosis, while systematically dissecting interactions among genomic background, modifier genes, environmental exposures, and additional factors such as the gut microbiome.

### 5.3. Limitations in the Clinical Application of Precision Therapies

Conventional DCM management, including pharmacological therapy, device-based interventions, and end-stage heart transplantation, aims to slow progression, alleviate symptoms, reduce hospitalization and mortality, and prevent SCD. However, these approaches rarely modify the underlying disease process, and many patients ultimately develop progressive heart failure. Given the marked heterogeneity of DCM, precision therapy is therefore particularly compelling. The goal is to deliver individualized interventions based on each patient’s molecular disease mechanisms, with the potential to delay or reverse progression. Because most pathogenic variants are individually rare, developing variant-specific therapies is costly and impractical. Accordingly, strategies targeting shared pathogenic pathways, such as sarcomeric dysfunction, inflammation, and fibrosis, or genotype-specific signaling cascades, including the *LMNA*–p38 MAPK axis, are more likely to achieve clinical feasibility.

Nevertheless, gene-directed precision therapies face substantial challenges, including optimization of delivery systems and dosing, packaging limits of AAV vectors (<5 kb), potential off-target effects, and risks of immunogenicity and organ toxicity (e.g., hepatic and neurological toxicity) [191]. Future efforts should prioritize safer and more efficient delivery platforms, such as next-generation AAV capsids with reduced immunogenicity, circular RNA [192], modified RNA [193], and exosome-based vectors [185]. Additionally, many studies remain confined to cellular or animal models that only partially recapitulate the complexity and heterogeneity of human disease. Integrating gene-editing technologies with stem cell-based approaches, including stem cell-derived gene therapies and humanized disease models, may provide more translational and durable solutions for DCM. 

## Figures and Tables

**Table 1:** Genetic Architecture and Core Molecular Mechanisms in DCM.

Genetic Category	Representative Genes	Protein Function	Core Molecular Mechanisms	Characteristic Clinical Features
Sarcomere	*TTN*, *MYH7*, *MYBPC3*	Sarcomeric structural and motor proteins	Altered sarcomere instability, impaired force generation	Variable penetrance, stress-sensitive phenotype, progressive systolic dysfunction
Nuclear, cytoskeletal and junctional	*LMNA*, *FLNC*, *DES*, *DSP*	Nuclear and cytoskeletal integrity, cell–cell adhesion, mechanosignaling	Nuclear instability, transcriptional dysregulation, DNA damage response activation, disrupted force transmission, cytoskeletal disorganization	Early-onset DCM, rapid progression, high arrhythmic risk, and/or skeletal myopathy
Calcium and ion handling	*SERCA2*, *RYR2*, *PLN*, *SCN5A*	Calcium cycling and electrical conduction	Calcium mishandling, electrical instability	DCM with conduction disease or arrhythmia-predominant phenotype
RNA splicing regulation	*RBM20*	Alternative splicing of cardiac genes	Aberrant splicing of sarcomeric and calcium-handling transcripts	Severe early-onset DCM, malignant ventricular arrhythmias
Mitochondrial dysfunction	*TAZ*, *NR2F2*, *PPARGC1A*	Mitochondrial structure or function	Reduce ATP production and increase oxidative stress	Severe DCM, premature death
Stress response and signaling	*BAG3*	Protein quality control and mechanosensing	Impaired proteostasis, defective stress adaptation	Progressive DCM, frequent heart failure progression
Proteostasis and protein aggregation	*DES*, *CRYAB*, *PLN*, *TTN*, *FLNC*	Protein quality control	Protein misfolding, aggregate toxicity, impaired degradation	Progressive DCM, fibrosis, arrhythmias

DCM: Dilated cardiomyopathy.

**Table 2:** Mechanism-Based Precision Therapeutic Strategies for DCM.

Pathogenic Mechanism	Therapeutic Target	Representative Strategies	Potential Target Population	Developmental Stage
Sarcomere dysfunction	Sarcomeric force generation and efficiency	Myosin modulators (e.g., omecamtiv mecarbil, danicamtiv)	Selected patients with sarcomeric or *TTN*-related DCM	Late-phase clinical and early implementation
Calcium handling abnormalities	Excitation–contraction coupling and calcium cycling	Modulation of calcium handling pathways	DCM with impaired calcium cycling and contractile reserve	Early clinical and translational
Inflammation and immune activation	Pro-inflammatory signaling pathways (e.g., IL-1β, NLRP3, JAK-STAT)	Cytokine inhibition and immune pathway modulation	Inflammatory-prone or CHIP-associated DCM	Early clinical and investigational
Myocardial fibrosis	Profibrotic signaling and extracellular matrix remodeling	RAAS blockade and emerging antifibrotic strategies	DCM with progressive remodeling and fibrotic burden	Established clinical
Metabolic remodeling	Mitochondrial function and substrate utilization	SGLT2 inhibitors and metabolic modulators	DCM with metabolic impairment and heart failure	Established clinical
Primary monogenic defects	Causal pathogenic variants	AAV-mediated gene replacement, ASOs- or RNA-based approaches	Monogenic DCM with defined molecular defects	Preclinical to early clinical
Advanced myocardial remodeling	Cardiomyocyte, inflammation and fibroblast targeting	iPSC-CMs, CAR-T-based inflammation and fibroblast modulation	Refractory or end-stage DCM	Experimental

DCM: Dilated cardiomyopathy; CHIP: Clonal hematopoiesis of indeterminate potential; RAAS: Renin–angiotensin–aldosterone system; SGLT2: Sodium–glucose cotransporter 2; AAV: Adeno-associated virus; ASOs: Antisense oligonucleotides; iPSC-CMs: Induced pluripotent stem cell-derived cardiomyocytes; CAR-T: Chimeric antigen receptor T cell therapy.

## Data Availability

No new data were created or analyzed in this study. Data sharing is not applicable to this article.

## Abbreviations

The following abbreviations are used in this manuscript:

DCM	Dilated cardiomyopathy
SCD	Sudden cardiac death
TTNtvs	truncating variants in *TTN*
HCM	Hypertrophic cardiomyopathy
SR	Sarcoplasmic reticulum
mtDNA	mitochondrial DNA
GWAS	Genome-wide association studies
AL	Immunoglobulin light-chain
PPCM	Peripartum cardiomyopathy
CH	Clonal Hematopoiesis
CHIP	CH of indeterminate potential
VAF	Variant allele frequency
MiRNAs	MicroRNAs
ECM	Extracellular matrix
ECG	Electrocardiogram
ICD	Implantable cardioverter-defibrillator
CMR	Cardiac magnetic resonance
LGE	Late gadolinium enhancement
EMB	Endomyocardial biopsy
CRT	Cardiac resynchronization therapy
LVAD	Left ventricular assist device
AAV	Adeno-associated virus
ASOs	Antisense oligonucleotides
iPSC-CMs	induced pluripotent stem cells-derived cardiomyocyte
CAR-T	Chimeric antigen receptor T cell
RAAS	Renin–angiotensin–aldosterone system
SGLT2	Sodium–glucose cotransporter 2
GDMT	Guideline-directed medical therapy
OM	Omecamtiv mecarbil
VDAC2	Voltage-dependent anion channel 2
iPSCs	induced pluripotent stem cells
